# 2-Amino-4-methyl­pyridinium 4-nitro­benzoate

**DOI:** 10.1107/S1600536810000693

**Published:** 2010-01-13

**Authors:** Madhukar Hemamalini, Hoong-Kun Fun

**Affiliations:** a X-ray Crystallography Unit, School of Physics, Universiti Sains Malaysia, 11800 USM, Penang, Malaysia

## Abstract

In the title salt, C_6_H_9_N_2_^+^·C_7_H_4_NO_4_^−^, the nitro group of the 4-nitro­benzoate anion is twisted by 7.66 (10)° from the attached ring. In the crystal structure, the 2-amino-4-methyl­pyridinium cations and 4-nitro­benzoate anions are linked *via* a pair of N—H⋯O hydrogen bonds to form a ribbon-like structure along the *c* axis. The ribbons are crosslinked into a three-dimensional framework by C—H⋯O hydrogen bonds.

## Related literature

For substituted pyridines, see: Pozharski *et al.* (1997 ▶); Katritzky *et al.* (1996 ▶). For bond-length data, see: Allen *et al.* (1987 ▶). For details of hydrogen bonding, see: Jeffrey & Saenger (1991 ▶); Jeffrey (1997 ▶); Scheiner (1997 ▶). For hydrogen-bond motifs, see: Bernstein *et al.* (1995 ▶). For the stability of the temperature controller used in the data collection, see: Cosier & Glazer (1986 ▶).
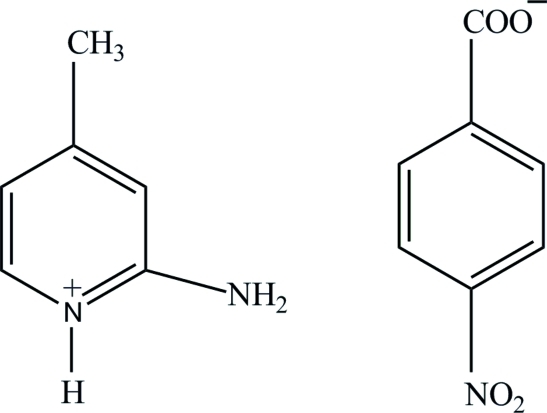


## Experimental

### 

#### Crystal data

C_6_H_9_N_2_^+^·C_7_H_4_NO_4_^−^
*M* *_r_* = 275.26
Monoclinic, 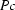

*a* = 10.5267 (2) Å
*b* = 5.0187 (1) Å
*c* = 12.2436 (3) Å
β = 92.194 (1)°
*V* = 646.36 (2) Å^3^
*Z* = 2
Mo *K*α radiation
μ = 0.11 mm^−1^
*T* = 100 K
0.49 × 0.28 × 0.16 mm

#### Data collection

Bruker SMART APEXII CCD area-detector diffractometer
Absorption correction: multi-scan (*SADABS*; Bruker, 2009 ▶) *T*_min_ = 0.949, *T*_max_ = 0.983
10644 measured reflections
2841 independent reflections
2390 reflections with *I* > 2σ(*I*)
*R*_int_ = 0.029

#### Refinement

*R*[*F* ^2^ > 2σ(*F* ^2^)] = 0.047
*wR*(*F* ^2^) = 0.124
*S* = 1.03
2841 reflections
222 parameters
2 restraints
H atoms treated by a mixture of independent and constrained refinement
Δρ_max_ = 0.44 e Å^−3^
Δρ_min_ = −0.30 e Å^−3^

### 

Data collection: *APEX2* (Bruker, 2009 ▶); cell refinement: *SAINT* (Bruker, 2009 ▶); data reduction: *SAINT*; program(s) used to solve structure: *SHELXS97* (Sheldrick, 2008 ▶); program(s) used to refine structure: *SHELXL97* (Sheldrick, 2008 ▶); molecular graphics: *SHELXTL* (Sheldrick, 2008 ▶); software used to prepare material for publication: *SHELXTL* and *PLATON* (Spek, 2009 ▶).

## Supplementary Material

Crystal structure: contains datablocks global, I. DOI: 10.1107/S1600536810000693/ci5013sup1.cif

Structure factors: contains datablocks I. DOI: 10.1107/S1600536810000693/ci5013Isup2.hkl

Additional supplementary materials:  crystallographic information; 3D view; checkCIF report

## Figures and Tables

**Table 1:** Hydrogen-bond geometry (Å, °)

*D*—H⋯*A*	*D*—H	H⋯*A*	*D*⋯*A*	*D*—H⋯*A*
N2—H1N2⋯O1^i^	0.93 (3)	2.55 (3)	3.254 (2)	134 (3)
N2—H1N2⋯O2^i^	0.93 (3)	1.78 (3)	2.688 (2)	167 (3)
N3—H1N3⋯O2^ii^	0.85 (4)	2.04 (4)	2.875 (2)	170 (4)
N3—H2N3⋯O1^i^	0.94 (3)	1.84 (3)	2.778 (2)	173 (3)
C3—H3*A*⋯O4^iii^	0.97 (2)	2.53 (2)	3.160 (2)	123 (2)
C6—H6*A*⋯O1^ii^	1.00 (2)	2.46 (2)	3.116 (2)	123 (3)
C7—H7*A*⋯O1^ii^	1.00 (3)	2.45 (3)	3.102 (2)	122 (2)
C9—H9*A*⋯O3^iv^	0.96 (3)	2.33 (3)	3.276 (3)	168 (3)
C13—H13*C*⋯O4^v^	0.96	2.55	3.335 (2)	139

Symmetry codes: (i) 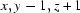; (ii) 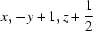; (iii) 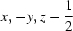; (iv) 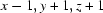; (v) 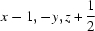.

**Fig. 1.**
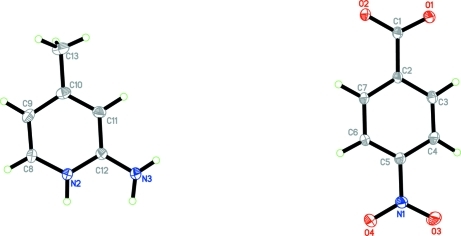
The asymmetric unit of the title compound, showing 50% probability displacement ellipsoids and the atom-numbering scheme.

**Fig. 2.**
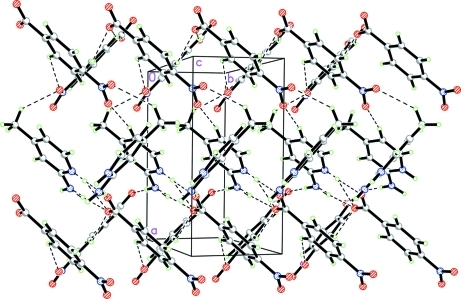
The crystal packing of the title compound, showing hydrogen-bonded (dashed lines) network.

## supplementary crystallographic information

### Comment

Pyridine and its derivatives play an important role in heterocyclic chemistry (Pozharski *et al.*, 1997; Katritzky *et al.*, 1996). Pyridine and its substituted derivatives are often involved in hydrogen-bond interactions (Jeffrey & Saenger, 1991; Jeffrey, 1997; Scheiner, 1997). Since our aim is to study some interesting hydrogen-bonding interactions, the crystal structure of the title compound is presented here.

The asymmetric unit of the title compound (Fig 1), contains a protonated 2-amino-4-methylpyridinium cation and a 4-nitrobenzoate anion. The 2-amino-4-methylpyridinium cation is planar, with a maximum deviation of 0.027 (1) Å for atom N3. The protonated N2 atom has lead to a slight increase in the C8—N2—C12 angle to  121.65 (14)°. In the 4-nitrobenzoate anion, the nitro group is twisted slightly from the ring with the dihedral angle between O3/O4/N1/C5 and C2-C7 planes being 7.66 (10)°. The bond lengths and angles are normal (Allen *et al.* 1987).

In the crystal packing (Fig. 2), the protonated N2 atom and 2-amino group (N3) is hydrogen-bonded to the carboxylate oxygen atoms (O1 and O2) via a pair of N—H···O hydrogen bonds leading to the formation of a *R*_2_^2^(8) ring (Bernstein *et al.* 1995). Furthermore, the crystal structure is stabilized by C—H···O hydrogen bonds to form a three-dimensional network.

### Experimental

A hot methanol solution (20 ml) of 2-amino-4-methylpyridine (27 mg, Aldrich) and 4-nitrobenzoic acid (42 mg, Merck) were mixed and warmed over a heating magnetic stirrer for a few minutes.  The resulting solution was allowed to cool slowly at room temperature and crystals of the title compound appeared after a few days.

### Refinement

The methyl H atoms were positioned geometrically [C–H = 0.96Å] and were refined using a riding model, with *U*_iso_(H) = 1.5*U*_eq_(C). A rotating group model was used for the methyl group. The remaining H atoms were located in a difference map and refined freely [N–H = 0.85 (4)–0.94 (3) Å and C–H = 0.95 (3)–1.00 (3) Å]. In the absence of significant anomalous scattering effects, 2841 Friedel pairs were merged.

### Crystal data

**Table:**

C_6_H_9_N_2_^+^·C_7_H_4_NO_4_^−^	*F*(000) = 288
*M**_r_* = 275.26	*D*_x_ = 1.414 Mg m^−^^3^
Monoclinic, *P**c*	Mo *K*α radiation, λ = 0.71073 Å
Hall symbol: P -2yc	Cell parameters from 3965 reflections
*a* = 10.5267 (2) Å	θ = 3.3–34.8°
*b* = 5.0187 (1) Å	µ = 0.11 mm^−^^1^
*c* = 12.2436 (3) Å	*T* = 100 K
β = 92.194 (1)°	Block, colourless
*V* = 646.36 (2) Å^3^	0.49 × 0.28 × 0.16 mm
*Z* = 2	

### Data collection

**Table:**

Bruker SMART APEXII CCD area-detector diffractometer	2841 independent reflections
Radiation source: fine-focus sealed tube	2390 reflections with *I* > 2σ(*I*)
graphite	*R*_int_ = 0.029
φ and ω scans	θ_max_ = 35.0°, θ_min_ = 1.9°
Absorption correction: multi-scan (*SADABS*; Bruker, 2009)	*h* = −16→16
*T*_min_ = 0.949, *T*_max_ = 0.983	*k* = −8→8
10644 measured reflections	*l* = −19→18

### Refinement

**Table:**

Refinement on *F*^2^	Primary atom site location: structure-invariant direct methods
Least-squares matrix: full	Secondary atom site location: difference Fourier map
*R*[*F*^2^ > 2σ(*F*^2^)] = 0.047	Hydrogen site location: inferred from neighbouring sites
*wR*(*F*^2^) = 0.124	H atoms treated by a mixture of independent and constrained refinement
*S* = 1.03	*w* = 1/[σ^2^(*F*_o_^2^) + (0.0808*P*)^2^] where *P* = (*F*_o_^2^ + 2*F*_c_^2^)/3
2841 reflections	(Δ/σ)_max_ = 0.001
222 parameters	Δρ_max_ = 0.44 e Å^−^^3^
2 restraints	Δρ_min_ = −0.30 e Å^−^^3^

### Special details

**Table:**

Experimental. The crystal was placed in the cold stream of an Oxford Cryosystems Cobra open-flow nitrogen cryostat (Cosier & Glazer, 1986) operating at 100.0 (1) k.
Geometry. All s.u.'s (except the s.u. in the dihedral angle between two l.s. planes) are estimated using the full covariance matrix. The cell s.u.'s are taken into account individually in the estimation of s.u.'s in distances, angles and torsion angles; correlations between s.u.'s in cell parameters are only used when they are defined by crystal symmetry. An approximate (isotropic) treatment of cell s.u.'s is used for estimating s.u.'s involving l.s. planes.
Refinement. Refinement of F^2^ against ALL reflections. The weighted R-factor wR and goodness of fit S are based on F^2^, conventional R-factors R are based on F, with F set to zero for negative F^2^. The threshold expression of F^2^ > 2σ(F^2^) is used only for calculating R-factors(gt) etc. and is not relevant to the choice of reflections for refinement. R-factors based on F^2^ are statistically about twice as large as those based on F, and R- factors based on ALL data will be even larger.

### Fractional atomic coordinates and isotropic or equivalent isotropic displacement parameters (Å^2^)

**Table:**

	*x*	*y*	*z*	*U*_iso_*/*U*_eq_	
O1	0.80158 (13)	0.6741 (3)	−0.02376 (11)	0.0242 (3)	
O2	0.71718 (12)	0.7465 (3)	0.13779 (10)	0.0199 (2)	
O3	1.22450 (16)	−0.2278 (4)	0.19930 (13)	0.0424 (5)	
O4	1.13745 (12)	−0.1978 (3)	0.35531 (10)	0.0223 (3)	
N1	1.14461 (14)	−0.1369 (3)	0.25872 (13)	0.0198 (3)	
C1	0.79450 (13)	0.6328 (3)	0.07603 (12)	0.0153 (3)	
C2	0.88627 (14)	0.4321 (3)	0.12679 (12)	0.0148 (3)	
C3	0.96760 (16)	0.2969 (4)	0.05869 (14)	0.0207 (3)	
C4	1.05331 (17)	0.1098 (4)	0.10127 (14)	0.0222 (3)	
C5	1.05432 (15)	0.0620 (3)	0.21297 (13)	0.0171 (3)	
C6	0.97482 (15)	0.1920 (3)	0.28289 (13)	0.0162 (3)	
C7	0.88970 (14)	0.3788 (4)	0.23830 (13)	0.0160 (3)	
N2	0.57997 (13)	0.1076 (3)	1.02192 (11)	0.0178 (3)	
N3	0.67843 (14)	0.0756 (3)	0.85722 (12)	0.0210 (3)	
C8	0.49010 (17)	0.2097 (4)	1.08697 (15)	0.0214 (3)	
C9	0.40877 (17)	0.4041 (4)	1.05116 (16)	0.0240 (3)	
C10	0.41950 (16)	0.5031 (3)	0.94309 (15)	0.0207 (3)	
C11	0.51011 (15)	0.3970 (3)	0.87848 (14)	0.0193 (3)	
C12	0.59201 (15)	0.1925 (3)	0.91783 (14)	0.0169 (3)	
C13	0.33051 (18)	0.7156 (4)	0.90181 (19)	0.0259 (4)	
H13A	0.3574	0.7791	0.8324	0.039*	
H13B	0.3308	0.8605	0.9530	0.039*	
H13C	0.2462	0.6439	0.8934	0.039*	
H3A	0.969 (2)	0.351 (5)	−0.017 (2)	0.023 (6)*	
H4A	1.110 (2)	0.010 (5)	0.055 (2)	0.020 (6)*	
H6A	0.977 (2)	0.176 (5)	0.364 (2)	0.025 (6)*	
H7A	0.833 (2)	0.476 (6)	0.289 (2)	0.028 (7)*	
H8A	0.479 (3)	0.152 (6)	1.160 (3)	0.045 (9)*	
H9A	0.347 (3)	0.490 (6)	1.095 (2)	0.036 (7)*	
H11A	0.519 (3)	0.464 (6)	0.804 (2)	0.029 (6)*	
H1N2	0.631 (3)	−0.026 (7)	1.052 (3)	0.043 (8)*	
H1N3	0.690 (3)	0.148 (8)	0.796 (3)	0.054 (10)*	
H2N3	0.726 (3)	−0.058 (7)	0.894 (3)	0.046 (8)*	

### Atomic displacement parameters (Å^2^)

**Table:**

	*U*^11^	*U*^22^	*U*^33^	*U*^12^	*U*^13^	*U*^23^
O1	0.0315 (7)	0.0287 (7)	0.0127 (5)	0.0121 (5)	0.0045 (5)	0.0033 (5)
O2	0.0231 (5)	0.0239 (6)	0.0131 (5)	0.0076 (5)	0.0048 (4)	0.0012 (4)
O3	0.0476 (10)	0.0597 (11)	0.0206 (7)	0.0367 (9)	0.0094 (7)	0.0023 (7)
O4	0.0258 (6)	0.0253 (7)	0.0161 (6)	0.0035 (5)	0.0024 (5)	0.0043 (5)
N1	0.0226 (6)	0.0214 (7)	0.0153 (6)	0.0055 (5)	0.0007 (5)	−0.0001 (5)
C1	0.0173 (6)	0.0164 (7)	0.0122 (7)	0.0004 (5)	0.0014 (5)	−0.0015 (5)
C2	0.0175 (6)	0.0162 (7)	0.0109 (6)	0.0009 (5)	0.0021 (5)	−0.0010 (5)
C3	0.0248 (8)	0.0263 (9)	0.0110 (7)	0.0063 (6)	0.0034 (6)	0.0006 (6)
C4	0.0254 (8)	0.0276 (9)	0.0139 (7)	0.0094 (7)	0.0054 (6)	−0.0019 (6)
C5	0.0172 (6)	0.0198 (8)	0.0143 (7)	0.0027 (5)	0.0009 (5)	−0.0005 (6)
C6	0.0177 (6)	0.0189 (8)	0.0121 (7)	0.0017 (5)	0.0023 (5)	0.0017 (5)
C7	0.0175 (6)	0.0191 (8)	0.0116 (6)	0.0032 (5)	0.0028 (5)	−0.0006 (5)
N2	0.0195 (6)	0.0195 (7)	0.0145 (6)	0.0048 (5)	0.0031 (5)	−0.0001 (5)
N3	0.0241 (7)	0.0228 (7)	0.0165 (6)	0.0065 (5)	0.0062 (5)	0.0045 (5)
C8	0.0245 (7)	0.0245 (8)	0.0155 (7)	0.0047 (6)	0.0045 (6)	−0.0026 (6)
C9	0.0229 (7)	0.0255 (9)	0.0238 (8)	0.0068 (6)	0.0050 (6)	−0.0045 (6)
C10	0.0194 (6)	0.0161 (7)	0.0263 (8)	0.0015 (5)	−0.0019 (6)	−0.0017 (6)
C11	0.0222 (7)	0.0160 (7)	0.0195 (7)	0.0018 (5)	−0.0006 (6)	0.0019 (5)
C12	0.0182 (6)	0.0167 (7)	0.0159 (7)	0.0009 (5)	0.0016 (5)	0.0010 (6)
C13	0.0237 (8)	0.0199 (8)	0.0336 (10)	0.0050 (6)	−0.0043 (7)	−0.0012 (7)

### Geometric parameters (Å, °)

**Table:**

O1—C1	1.244 (2)	N2—C8	1.360 (2)
O2—C1	1.2675 (19)	N2—H1N2	0.93 (3)
O3—N1	1.221 (2)	N3—C12	1.332 (2)
O4—N1	1.227 (2)	N3—H1N3	0.85 (4)
N1—C5	1.474 (2)	N3—H2N3	0.94 (3)
C1—C2	1.513 (2)	C8—C9	1.359 (3)
C2—C7	1.390 (2)	C8—H8A	0.95 (3)
C2—C3	1.394 (2)	C9—C10	1.422 (3)
C3—C4	1.390 (3)	C9—H9A	0.96 (3)
C3—H3A	0.96 (3)	C10—C11	1.370 (2)
C4—C5	1.388 (2)	C10—C13	1.495 (3)
C4—H4A	0.98 (2)	C11—C12	1.413 (2)
C5—C6	1.383 (2)	C11—H11A	0.98 (3)
C6—C7	1.394 (2)	C13—H13A	0.96
C6—H6A	0.99 (3)	C13—H13B	0.96
C7—H7A	1.00 (3)	C13—H13C	0.96
N2—C12	1.354 (2)		
			
O3—N1—O4	123.39 (16)	C8—N2—H1N2	116.1 (19)
O3—N1—C5	118.38 (16)	C12—N3—H1N3	116 (2)
O4—N1—C5	118.21 (14)	C12—N3—H2N3	114.0 (19)
O1—C1—O2	125.08 (16)	H1N3—N3—H2N3	130 (3)
O1—C1—C2	116.94 (13)	C9—C8—N2	121.68 (17)
O2—C1—C2	117.98 (14)	C9—C8—H8A	115.0 (19)
C7—C2—C3	120.02 (15)	N2—C8—H8A	123.4 (19)
C7—C2—C1	121.57 (13)	C8—C9—C10	118.65 (15)
C3—C2—C1	118.40 (14)	C8—C9—H9A	125.2 (17)
C4—C3—C2	120.61 (16)	C10—C9—H9A	116.0 (18)
C4—C3—H3A	121.0 (14)	C11—C10—C9	118.90 (15)
C2—C3—H3A	118.0 (14)	C11—C10—C13	121.55 (17)
C5—C4—C3	117.84 (15)	C9—C10—C13	119.53 (17)
C5—C4—H4A	120.0 (14)	C10—C11—C12	120.97 (16)
C3—C4—H4A	122.1 (14)	C10—C11—H11A	119.6 (16)
C6—C5—C4	123.09 (15)	C12—C11—H11A	119.4 (16)
C6—C5—N1	118.73 (15)	N3—C12—N2	118.43 (15)
C4—C5—N1	118.17 (15)	N3—C12—C11	123.42 (16)
C5—C6—C7	118.03 (15)	N2—C12—C11	118.13 (15)
C5—C6—H6A	126.2 (14)	C10—C13—H13A	109.5
C7—C6—H6A	115.6 (14)	C10—C13—H13B	109.5
C2—C7—C6	120.40 (14)	H13A—C13—H13B	109.5
C2—C7—H7A	121.4 (16)	C10—C13—H13C	109.5
C6—C7—H7A	118.2 (16)	H13A—C13—H13C	109.5
C12—N2—C8	121.65 (14)	H13B—C13—H13C	109.5
C12—N2—H1N2	122.2 (19)		
			
O1—C1—C2—C7	177.48 (16)	N1—C5—C6—C7	−179.78 (15)
O2—C1—C2—C7	−2.2 (2)	C3—C2—C7—C6	0.5 (2)
O1—C1—C2—C3	−3.3 (2)	C1—C2—C7—C6	179.72 (15)
O2—C1—C2—C3	177.03 (16)	C5—C6—C7—C2	−0.4 (2)
C7—C2—C3—C4	−0.5 (3)	C12—N2—C8—C9	−0.7 (3)
C1—C2—C3—C4	−179.77 (17)	N2—C8—C9—C10	−0.5 (3)
C2—C3—C4—C5	0.4 (3)	C8—C9—C10—C11	0.8 (3)
C3—C4—C5—C6	−0.3 (3)	C8—C9—C10—C13	179.63 (18)
C3—C4—C5—N1	179.75 (17)	C9—C10—C11—C12	0.0 (2)
O3—N1—C5—C6	−171.50 (18)	C13—C10—C11—C12	−178.76 (16)
O4—N1—C5—C6	6.8 (2)	C8—N2—C12—N3	−177.11 (16)
O3—N1—C5—C4	8.5 (3)	C8—N2—C12—C11	1.5 (2)
O4—N1—C5—C4	−173.20 (17)	C10—C11—C12—N3	177.40 (17)
C4—C5—C6—C7	0.2 (3)	C10—C11—C12—N2	−1.2 (2)

### Hydrogen-bond geometry (Å, °)

**Table:**

*D*—H···*A*	*D*—H	H···*A*	*D*···*A*	*D*—H···*A*
N2—H1N2···O1^i^	0.93 (3)	2.55 (3)	3.254 (2)	134 (3)
N2—H1N2···O2^i^	0.93 (3)	1.78 (3)	2.688 (2)	167 (3)
N3—H1N3···O2^ii^	0.85 (4)	2.04 (4)	2.875 (2)	170 (4)
N3—H2N3···O1^i^	0.94 (3)	1.84 (3)	2.778 (2)	173 (3)
C3—H3A···O4^iii^	0.97 (2)	2.53 (2)	3.160 (2)	123 (2)
C6—H6A···O1^ii^	1.00 (2)	2.46 (2)	3.116 (2)	123 (3)
C7—H7A···O1^ii^	1.00 (3)	2.45 (3)	3.102 (2)	122 (2)
C9—H9A···O3^iv^	0.96 (3)	2.33 (3)	3.276 (3)	168 (3)
C13—H13C···O4^v^	0.96	2.55	3.335 (2)	139

Symmetry codes: (i) *x*, *y*−1, *z*+1; (ii) *x*, −*y*+1, *z*+1/2; (iii) *x*, −*y*, *z*−1/2; (iv) *x*−1, *y*+1, *z*+1; (v) *x*−1, −*y*, *z*+1/2.
